# Ablation of ventricular tachycardia using state-of-the-art preprocedural imaging, magnetic-based 3-dimensional mapping, and ultra-low-temperature cryoablation technology

**DOI:** 10.1016/j.hrcr.2022.02.013

**Published:** 2022-03-04

**Authors:** Max Liebregts, Vincent F. van Dijk, Lucas V.A. Boersma, Jippe C. Balt

**Affiliations:** ∗ Department of Cardiology, St. Antonius Hospital Nieuwegein, Nieuwegein, The Netherlands; † Department of Cardiology, Amsterdam UMC, Amsterdam, The Netherlands

**Keywords:** Ventricular tachycardia, Magnetic resonance imaging, Preprocedural imaging, Electroanatomical mapping, Cryoablation

**Key Teaching Points**

• Preprocedural imaging with ADAS 3D software (Galgo Medical S.L., Barcelona, Spain) can create a 3-dimensional model of the heart derived from magnetic resonance imaging and computed tomography.
• Ensite X’s VoXel Mode (St Jude Medical, St Paul, MN) can create an electroanatomical map using magnetic-based coordinates.
• The Adagio Medical VT Cryoablation System (Adagio Medical, Inc, Laguna Hills, CA) uses “near-critical” nitrogen refrigerant (T ≈ -196°C) and has been shown to produce durable transmural lesions.

## Introduction

We describe a case of ultra-low-temperature cryoablation for treatment of monomorphic ventricular tachycardia (VT) performed as part of the first-in-human study (Cryocure-VT, NCT#04893317).

## Case report

**Figure 1:**
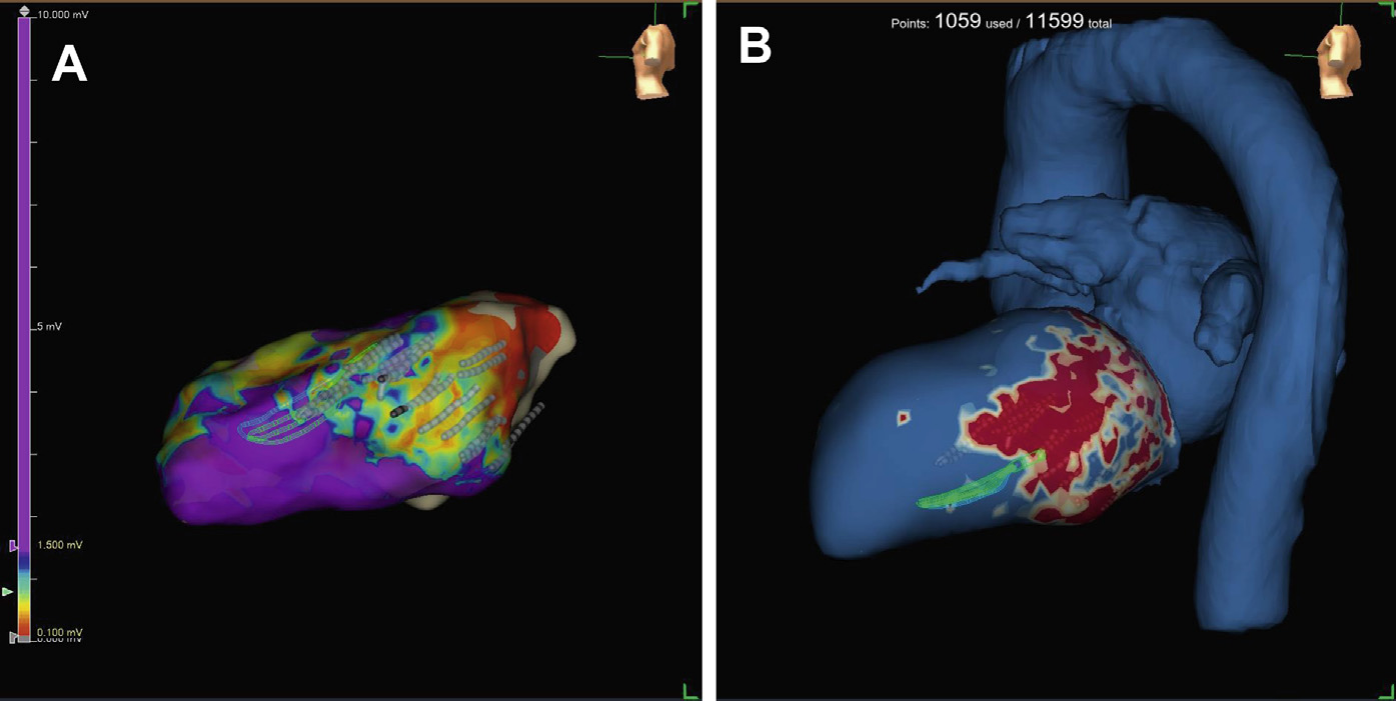
**A:** Voltage map of the left ventricle using Ensite X’s VoXel mode (St Jude Medical, St Paul, MN) (bipolar voltage window: 0.1–1.5 mV). The Advisor HD Grid mapping catheter (Abbott Medical, Inc, Minneapolis, MN) is shown in yellow. Shadows of the Adagio ventricular tachycardia catheter (Adagio Medical, Inc, Laguna Hills, CA) at the different ablation sites are shown in gray. **B:** Three-dimensional model of the heart with the different layers of the left ventricle derived from magnetic resonance imaging (0–10% endocardial layer shown) and the remaining heart chambers and great vessels derived from computed tomography. Red indicates dense scar, yellow indicates border zone. The HD Grid catheter is shown in yellow and shadows of the Adagio catheter at the different ablation sites are shown in gray.

**Figure 2:**
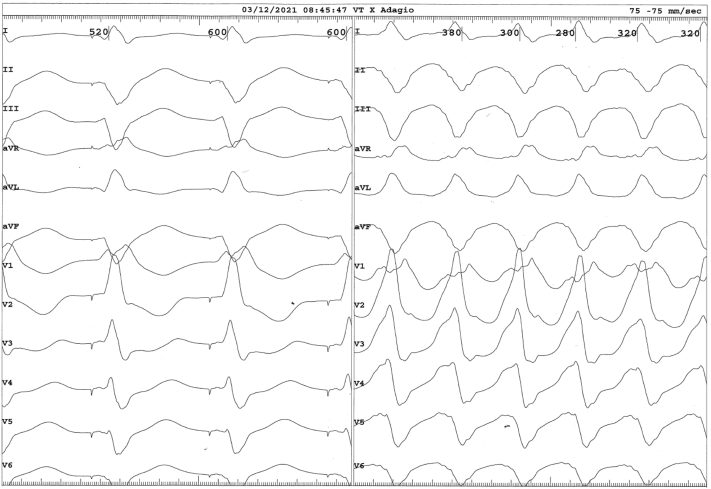
Left panel: Pace mapping at the mid segment of the inferior border of the lateral infarction. Right panel: A 12-lead electrocardiogram of the ventricular tachycardia showing a 12/12 match.

**Figure 3:**
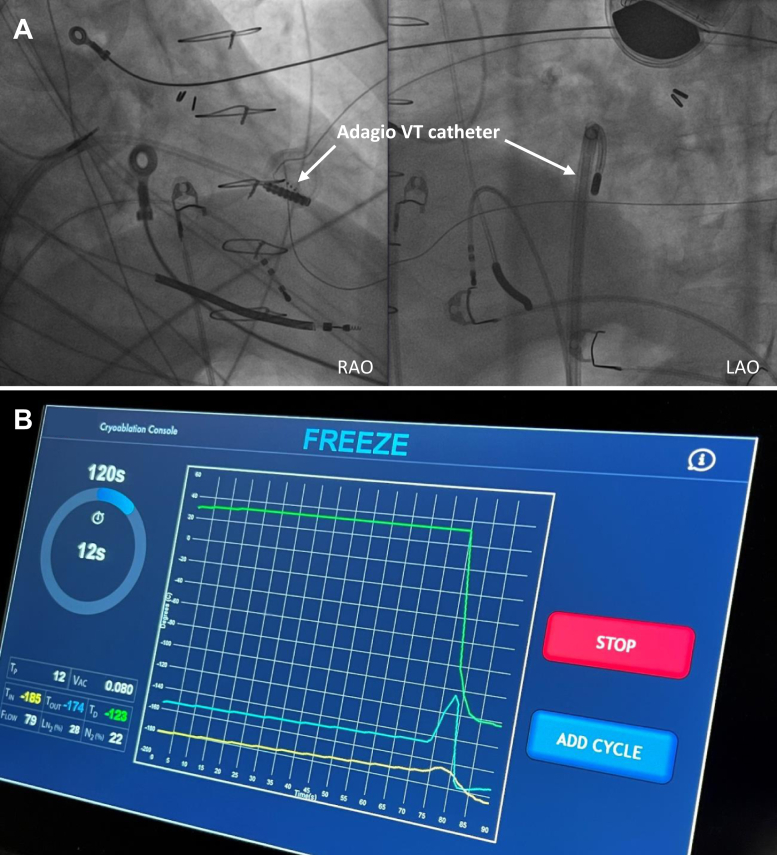
**A:** Fluoroscopic images of the ablation procedure showing the Adagio ventricular tachycardia catheter (Adagio Medical, Inc, Laguna Hills, CA) in left anterior oblique (LAO) and right anterior oblique (RAO) view. **B:** Adagio cryoablation console. The yellow line in the graph indicates the temperature going into the catheter, the blue line indicates the temperature going out of the catheter, and the green line indicates the temperature at the cryoablation element on the distal end of the catheter.

The patient was a 75-year-old man who was referred for VT ablation because of incessant VTs despite treatment with amiodarone. The patient had undergone an emergency coronary artery bypass grafting following a complicated percutaneous coronary intervention in 2014 and an implantable cardioverter-defibrillator was implanted for secondary prevention in February 2021. A magnetic resonance imaging (MRI) with whole-heart isotropic-resolution 3-dimensional (3D) late gadolinium enhancement sequence was performed before implantable cardioverter-defibrillator implantation and showed a transmural lateral infarction. MRI images were integrated with computed tomography images using ADAS 3D software (Galgo Medical S.L., Barcelona, Spain), resulting in a 3D model with myocardial scar shown in the different layers of the left ventricle, derived from the MRI, combined with the remaining heart chambers and great vessels, derived from the computed tomography, as landmarks.^1^ The model was uploaded to an Ensite X EP System (St Jude Medical, St Paul, MN) at the start of the procedure (Figure 1B). Subsequently a voltage map was created using the Advisor HD Grid mapping catheter (Abbott Medical, Inc, Minneapolis, MN) and Ensite VoXel Mode, which collects modeling data using magnetic-based coordinates (Figure 1A).^2^ Ventricular late potentials coincided with the lateral myocardial infarction and were annotated on the voltage map. A VT with cycle length 330 ms was induced with a drivetrain at 600 ms with S2 310 ms and S3 290 ms. The 12-lead electrocardiogram showed evidence of an exit site at the mid segment of the inferior border of the lateral infarction. Activation mapping was not possible owing to hemodynamic instability and the VT was terminated by electrical cardioversion. Nevertheless, during 30 seconds, diastolic potentials were recorded close to the putative VT isthmus. Pace mapping at the presumed location showed a 12/12 match (Figure 2). Ablation was performed via transseptal approach using the Adagio Medical VT Cryoablation System (Adagio Medical, Inc, Laguna Hills, CA). The system consists of a cryoablation console (Figure 3B) and a 9F bidirectional deflectable VT catheter with 15-mm-long, 8-electrode cryoablation element (Figure 3A). Liquid nitrogen circulates through a cooling system from the console to the cryoablation element on the distal end of the catheter. This enables the operator to perform ablation with ultralow temperatures with a theoretical minimum of -196°C, which has been shown to produce durable transmural lesions in animal models.^3^^,^^4^ The catheter was visualized by the Ensite X EP System by localizing its electrodes. Two 2-minute freezes (with 1-minute thaw in between) were performed at 9 ablation sites (as shown by catheter shadows in Figure 1), for a total freeze time of 36 minutes, covering the lateral infarcted area and all areas with ventricular late potentials. Cryoadhesion during the applications resulted in stable catheter positioning. Afterward the clinical VT could not be induced; there was only a polymorphic VT after a drivetrain at 600 ms with S2, S3, and S4 at 300 ms, which was not pursued.

## Conclusion

No complications occurred and no adverse events were reported during the use of the Adagio VT Cryoablation System.
